# 

**Published:** 1897-11-27

**Authors:** 


### EARLY EDITION.

**Notices of Births, Deaths, and Marriages** are inserted in this column at a charge of 2s. 6d. for an announcement not exceeding four lines.

### NOTICE TO CORRESPONDENTS.

All MS., Letters, Books for Review, and other matters intended for the Editor should be addressed THE EDITOR, "THE HOSPITAL," THE HOSPITAL BUILDING, 28 & 29, SOUTHAMPTON STREET, STRAND, W.C.

The Editor cannot undertake to return rejected MS., even when accompanied by stamped directed envelope.

**Notice to Advertisers and Subscribers.**

All Advertisements, Orders for Copies of THE HOSPITAL, and Business Communications should be addressed to **THE MANAGER, (Not to The Editor),** THE HOSPITAL, The Hospital Building, 28 & 29, Southampton Street, Strand, London, W.C.

The Cost of Subscription, post free, is as follows:—Per week, 2½d.; per quarter, 2s. 8d.; per half-year, 5s. 3d.; per annum, 10s. 6d. Foreign:—Per annum, 15s. 2d.; payable, in all cases, in advance.

**NOTICE.—Vol. XXII. of "The Hospital" (April, 1897, to September, 1897), in handsome cloth binding, is now on sale at the Office of this Journal, price 7s. 6d. Cases for binding the Numbers contained in this Volume, 1s. 6d. Index to Vol. XXII., 2½d., post free.**

**The Monthly Part for December will be ready on December 1st, Price 6d., by post 8d.**

### BOOKS RECEIVED.

A. D. Innes and Co.

"Inspector-General Sir James Ranald Martin, C.B., F.R.S." By Surgeon-General Sir Joseph Fayrer, Bart.

John Hogg.

"Cookery for Invalids." By Lizzie Heritage.

James Nisbet and Co.

"Health in Africa." A Medical Handbook for Travellers. By D. Kerr Cross, M.B., C.M., F.R.G.S. With an introduction by Sir Harry Johnston, K.C.B.

"Localisation of Headache and Sick Headache." By H. Bendelack Hewetson.

Adam and Charles Black.

"Black's Guide to Bath and Bristol." Edited by A. R. Hope Moncrieff.

**Periodicals and Pamphlets Received.**—Electrical Review, Therapist, Local Government Journal, School Board Chronicle, Woman's Signal, London; Public Health Progress in the Queen's Reign, by T. Ridley Bailey, M.D.

### THE STUDENT OF MEDICINE.

### PASS LIST.

### University of London.

M.B. Examination: October, 1897.

First Division.—C. W. Alford, Middlesex Hospital; M. Dixon, B.Sc., University College; Brennan Dyball, St. Thomas's Hospital; J. L. Jones, University College; W. P. Nicol, Mason College; A. W. Sikes, B.Sc., St. Thomas's Hospital; F. H. Thiele, B.Sc., University College; E. J. Toye, B.Sc., St. Bartholomew's Hospital; A. G. Wilson, London Hospital.

Second Division.—E. W. Adams, University College, Sheffield; Louisa Garrett Anderson, London School of Medicine for Women; W. R. Battye, B.Sc.. University College; C. H. Benham, University College; H. C. P. Bennett, St. Bartholomew's Hospital; S. H. Berry, Charing Cross Hospital; J. Broadley, Yorkshire College; H. W. Bruce, Guy's Hospital; A. Burn, St. Mary's Hospital; J. E. G. Calverley, St. Bartholomew's Hospital; C. C. Clarkson, University College; A. S. Cobbledick, Bristol Medical School; E. Coleman, Guy's Hospital; Mary Frances Cornford, Royal Free Hospital; S. Cornish, St. Bartholomew's Hospital; T. V. Cunliffe, Owens College and Manchester Royal Infirmary; W. A. Dewhirst, Yorkshire College; M. B. Douie, London School of Medicine and Royal Free Hospital; W. E. N. Dunn, St. Bartholomew's Hospital; C. Dykes, University College; C. H. Fagge, Guy's Hospital; E. G. L. Goffe, University College; C. P. Harris, London Hospital; Alice Mary Hawker, London School of Medicine for Women; C. R. Hodgson, Guy's Hospital; A. L. Home, St. Thomas's Hospital; G. Hutcheson, London Hospital; A. E. Jerman, Westminster Hospital; Beatrice Knowles, London School of Medicine and Royal Free Hospital; H. Leader, Guy's Hospital; G. K. Levick, Guy's Hospital; R. D. Maxwell, London Hospital; R. W. Mayston, Guy's Hospital; C. H. Melland, Owens College and Manchester Royal Infirmary; W. T. Milton, Guy's Hospital; H. P. Noble, Middlesex Hospital; J. H. Phipps, Owens College, Manchester Royal Infirmary, and Guy's Hospital; R. W. C. Pierce, B.Sc., St. Thomas's Hospital; G. E. Richmond, B.A., B.Sc., Guy's Hospital; J. T. Roberts, Guy's Hospital; F. W. Robertson, St. Bartholomew's Hospital: T. O'Neill Roe, University College; J. P. Scatchard, St. Thomas's Hospital; J. Shardlow, University College; Mary Nona Sharman, Royal Free Hospital; E. B. Sherlock, B.Sc., Westminster Hospital; J. E. Smith, University College, Liverpool; L. A. Smith, London Hospital; H. J. Starling, Guy's Hospital; E. S. Stork, University College; P. C. E. Tribe, King's College; W. P. Walker, Owens College and Manchester Royal Infirmary; R. J. Warrington, Owens College and Manchester Royal Infirmary; Bertha Margaret Webb, London School of Medicine and Royal Free Hospital; P. G. S. Williams, University College; T. H. Woodfield, St. Bartholomew's Hospital.

